# Emergency general surgery in Rwandan district hospitals: a cross-sectional study of spectrum, management, and patient outcomes

**DOI:** 10.1186/s12893-017-0323-x

**Published:** 2017-12-01

**Authors:** Christophe Mpirimbanyi, Alexandre Nyirimodoka, Yihan Lin, Bethany L. Hedt-Gauthier, Jackline Odhiambo, Theoneste Nkurunziza, Joaquim M. Havens, Jack Omondi, Emile Rwamasirabo, Faustin Ntirenganya, Gabriel Toma, Joel Mubiligi, Scheilla Bayitondere, Robert Riviello

**Affiliations:** 1 College of Medicine and Health Sciences, University of Rwanda, P.O. Box 3286, Kigali, Rwanda; 2 Department of Global Health and Social Medicine, Harvard Medical School, Boston, USA; 3 Partners In Health/Inshuti Mu Buzima, Kigali, Rwanda; 4 Partners In Health, Boston, USA; 5 Center for Surgery and Public Health, Brigham and Women’s Hospital, Boston, USA; 6 Rwandan Surgical Society, Kigali, Rwanda; 7 Ministry of Health, Kigali, Rwanda

**Keywords:** Acute care surgery, Emergency general surgery, Soft tissue infections, Acute abdominal conditions, Africa, Complicated hernia

## Abstract

**Background:**

Management of emergency general surgical conditions remains a challenge in rural sub-Saharan Africa due to issues such as insufficient human capacity and infrastructure. This study describes the burden of emergency general surgical conditions and the ability to provide care for these conditions at three rural district hospitals in Rwanda.

**Methods:**

This retrospective cross-sectional study included all patients presenting to Butaro, Kirehe and Rwinkwavu District Hospitals between January 1st 2015 and December 31st 2015 with emergency general surgical conditions, defined as non-traumatic, non-obstetric acute care surgical conditions. We describe patient demographics, clinical characteristics, management and outcomes.

**Results:**

In 2015, 356 patients presented with emergency general surgical conditions. The majority were male (57.2%) and adults aged 15–60 years (54.5%). The most common diagnostic group was soft tissue infections (71.6%), followed by acute abdominal conditions (14.3%). The median length of symptoms prior to diagnosis differed significantly by diagnosis type (*p* < 0.001), with the shortest being urological emergencies at 1.5 days (interquartile range (IQR):1, 6) and the longest being complicated hernia at 17.5 days (IQR: 1, 208). Of all patients, 54% were operated on at the district hospital, either by a general surgeon or general practitioner. Patients were more likely to receive surgery if they presented to a hospital with a general surgeon compared to a hospital with only general practitioners (75% vs 43%, *p* < 0.001). In addition, the general surgeon was more likely to treat patients with complex diagnoses such as acute abdominal conditions (33.3% vs 4.1%, *p* < 0.001) compared to general practitioners. For patients who received surgery, 73.3% had no postoperative complications and 3.2% died.

**Conclusion:**

While acute abdominal conditions are often considered the most common emergency general surgical condition in sub-Saharan Africa, soft tissue infections were the most common in our setting. This could represent a true difference in epidemiology in rural settings compared to referral facilities in urban settings. Patients were more likely to receive an operation in a hospital with a general surgeon as opposed to a general practitioner. This provides evidence to support increasing the surgical workforce in district hospitals in order to increase surgical availability for patients.

## Background

As of 2015, an estimated 5 billion people do not have access to timely, safe, and affordable surgical and anesthesia care [1]. Improving infrastructure and increasing human resources to provide surgery in district hospitals is critical to address this gap [2].Both the Lancet Commission on Global Surgery and the World Bank’s “Essential Surgery Package” recommend that basic emergency and essential general surgical procedures should be available at the district hospital level [1, 3]. However, performing these procedures at district hospitals is often a challenge. Surgical delivery is often complicated by inadequate diagnostic tools, inadequately trained personnel, and insufficient infrastructure for resuscitation and surgery [4]. Combined with patient delays in presentation, these factors contribute to poor patient outcomes [5].

Currently, there are limited studies in sub-Saharan Africa assessing the burden of emergency general surgical conditions, defined in this paper as non-traumatic, non-obstetric acute surgical conditions, and the care provided for these patients at rural district hospitals. In Rwanda, a patient with an emergency general surgical condition usually first presents to a health center for care and is referred to the district hospital if surgery is needed. While, 82.5% of operations occur in the district hospitals, most of these are cesarean sections [6].For non-obstetric conditions, most patients present with trauma (42.6%), infection (22.5%), and general surgery including abdominal surgical conditions, hernias, soft tissues and skin conditions (21.1%), surgically resectable cancers (10.5%), urology (2.0%) and congenital defects (1.3%) [7]. Very few of the 42 district hospitals can provide emergency general surgery for patients presenting with these general surgical conditions or orthopedic conditions [6].

At the district hospital, patients undergo an operation if there is a provider with adequate experience as well as appropriate equipment and infrastructure at the facility. Typically, general practitioners perform minor procedures and some major procedures such as caesarian sections. Anesthesia care is provided by anesthesia technicians and peri-operative nursing care by non-specialized nursing staff [6]. If equipment and trained staff are not available, the patient is referred to a tertiary hospital in an urban location. Patients with health insurance pay 10–15% of the hospital costs, with the remainder being covered by their insurance provider, while those without insurance are responsible for all their medical expenses. This study describes clinical presentations, management, and outcomes associated with emergency general surgical conditions presenting at three district hospitals in rural Rwanda. The study aims to improve the understanding of existing capacities and current gaps in caring for these patients in rural sub-Saharan Africa.

## Methods

### Study setting

This study was conducted at three of the 42 district hospitals in Rwanda: Butaro District Hospital located in the Northern Province with a catchment population of 340,000 people and Kirehe and Rwinkwavu District Hospitals located in the Eastern Province with catchment populations of 292,215 and 265,000, respectively. Care at these hospitals is led by the Ministry of Health with support from Partners In Health/Inshuti Mu Buzima (PIH/IMB), a US-based non-governmental organization. In 2015, each of the hospitals had two operating theaters. Butaro had one general surgeon on staff but Kirehe and Rwinkwavu had no surgeons. Occasionally, these hospitals, particularly Butaro, received external missions that provide surgical care. In addition, PIH/IMB provided economic and social support to select patients who had challenges accessing care.

### Study design and population

This retrospective cross-sectional study included patients who presented between 01 January and 31 December 2015 with an emergency general surgical condition at Butaro, Kirehe and Rwinkwavu District Hospitals and were admitted to the emergency and surgical wards of these district hospitals for management. We excluded patients admitted in other hospital wards, and those transferred directly to tertiary care with only names documented in the district hospital emergency logbook without available and completed medical files. We defined emergency general surgical conditions as non-traumatic and non-obstetric acute care surgical conditions. While there are more conditions that meet this definition, in this paper we focused on the most common conditions of:1) acute abdominal conditions (bowel obstruction, perforation, cholecystitis, appendicitis, viscous strangulation, and other unspecified peritonitis), 2) complicated hernias (incarceration, obstruction, and strangulation), 3) soft tissue infections (abscess, cellulitis, pyomyositis, extremity gangrene, and deep tissue infections), 4) urological emergencies (acute urinary retention and testicular torsion), and5) thoracic emergencies (empyema, massive pleural effusion, and non-traumatic pneumothorax).

### Data collection and analysis

Data was extracted from hospital admission registers, patient files and operating room logbooks from the three district hospitals. Trained data collectors used a predesigned tool to collect patient data on paper forms. Data was entered into an electronic Access database and analyzed using Stata v13.0 (College Station, TX: StataCorp LP). We described patient demographics, clinical characteristics, management, and outcomes using frequencies and percents for categorical data, and median and interquartile ranges for continuous data. We assessed the relationship between district hospital and whether the patient received surgery. We then compared the diagnosis type, surgical intervention, and treatment outcome by whether a patient was operated on by general surgeon or a general practitioner. We used Fisher’s exact test for categorical variables and Kruskal-Wallis Rank Sum test for continuous variables.

## Results

In 2015, 356 patients presented with emergency general surgical conditions at the three district hospitals. The majority of these patients were male (57.2%, *n* = 202 out of 353) and aged 15–60 years (54.5%, *n* = 186 out of 341) (Table 1). The distribution of patients was relatively even across hospitals (Butaro: *n* = 108, 30.3%; Kirehe: *n* = 131, 36.8%; Rwinkwavu: *n* = 117, 32.9%). Among the 237 patients who had health insurance status recorded, 95.4% (*n* = 226) were insured.

**Table 1:** Demographic characteristics of patients with emergency general surgical conditions at three rural district hospitals in Rwanda (*N* = 356)

Demographics	*n*	Percent
Sex (*n* = 353)		
Male	202	57.2
Female	151	42.8
Age (*n* = 341)		
< 5 years	47	13.8
5–15 years	60	17.6
15–40 years	115	33.7
40–60 years	71	20.8
> 60 years	48	14.1
Hospital		
Butaro	108	30.3
Kirehe	131	36.8
Rwinkwavu	117	32.9
Health insurance status (*n* = 237)		
With insurance	226	95.4
Without insurance	11	4.6

The most common primary diagnoses were soft tissue infections (71.6%, *n* = 255) followed by acute abdominal conditions (14.3%, *n* = 51), complicated hernias (7.9%, *n* = 28), urological emergencies (5.3%, *n* = 19), and thoracic emergencies (0.9%, *n* = 3) (Table 2). Within soft tissue infections, abscesses(52.6%, *n* = 134) followed by pyomyositis (17.7%, *n* = 45) were the most common diagnoses. Bowel obstruction (*n* = 23, 45.1%) followed by volvulus (n = 10, 19.6%) were the most common type of acute abdominal conditions. Of the 274 patients with duration of symptoms recorded, 54 (19.7%) presented within 3 days of symptom onset and the majority (*n* = 172, 62.8%) presented after 7 days or more. The duration of symptoms prior to presentation differed by diagnosis type as follows: complicated hernias had a median duration of 17.5 days (interquartile range (IQR) =1, 208), soft tissue infections was 10 days (IQR = 6, 21), acute abdominal conditions was four days (IQR = 2, 7), and urological emergencies was 1.5 days (IQR = 1, 6) (*p* < 0.001). Among the 141 patients with prior surgical history documented, 25 (17.7%) had a history of previous surgery and for the 222 with past medical history recorded, 52 (23.4%) had at least one underlying medical illness such as hypertension, diabetes, HIV, or psychosis.

**Table 2:** Diagnoses and clinical characteristics of emergency general surgical patients (*N* = 356)

	n	Percent
Emergency surgical conditions diagnosis		
Soft tissue infections	255	71.6
Abscess	134	52.6
Pyomyositis	45	17.7
Unspecified deep tissue infections	41	16.1
Cellulitis	21	8.2
Necrotizing fasciitis	8	3.1
Extremity gangrene	6	2.3
Acute abdominal conditions	51	14.3
Bowel obstruction	23	45.1
Volvulus	10	19.6
Bowel perforation	6	11.8
Unspecified peritonitis	6	11.8
Appendicitis	5	9.8
Cholecystitis	1	1.9
Complicated hernia	28	7.9
Incarcerated	15	53.6
Obstruction/strangulation	13	46.4
Urological emergencies	19	5.3
Acute urinary retention	13	68.4
Testicular torsion	6	31.6
Thoracic emergencies	3	0.9
Pneumothorax	2	66.7
Empyema	1	33.3
Duration of symptoms (*n* = 274)		
< 3 days	54	19.7
3–6 days	48	17.5
7–29 days	108	39.4
≥ 30 days	64	23.4
Hypotensive at admission(Systolic Blood Pressure < 90 mmHg)(n = 226)	8	3.5
History of prior surgery (*n* = 141)	25	17.7
Underlying medical illness^a^ (*n* = 222)	52	23.4

^a^Includes: Hypertension, diabetes, cardiac, stroke, kidney disease, HIV, liver disease, hematological disorder, psychiatric diagnosis

Non-surgical treatment included antibiotics (93.3%, *n* = 332), wound dressing (49.7%, *n* = 177), insertion of urethral catheter (10.1%, *n* = 36), insertion of nasogastric tube (9.6%, *n* = 34), and blood transfusion (2%, *n* = 7) (Table 3). Approximately half of patients (*n* = 188, 54.0%) underwent an operation at the district hospital, 185(52.0%) received surgery and non-surgical management and three patients (0.8%) received surgery alone. Another 143 patients (43.5%) received non-surgical treatment and for 13 patients (3.7%), we found no indication of receipt of surgery or non-surgical management. For the 188 patients who received surgery, 81 (43.1%) of these operations occurred at Butaro, compared to 59 (30.8%) at Rwinkwavu, and 49 (26.1%) at Kirehe (Table 3). Patients presenting at Butaro District Hospital were significantly more likely to receive surgery compared to the other two hospitals (75%vs 43%, *p* < 0.001).For the 110 patients with type of anesthesia recorded, the most common was spinal/regional anesthesia (35.5%, *n* = 39) followed by general anesthesia (30.0%, *n* = 33), sedation (29.1%, *n* = 32) and local anesthesia (5.4%, *n* = 6). Type of surgical provider was recorded for 122 patients, with74 (60.7%) operated on by a general practitioner and 48(39.3%) by a general surgeon. The most common surgical procedure was incision and drainage (61.3%, *n* = 114 out of 186), followed by laparotomy (10.8%, *n* = 20), debridement (9.1%, *n* = 17), hernia repair (5.4%, *n* = 10), amputation (4.8%, *n* = 9) and suprapubic catheterization (2.7%, *n* = 5).

**Table 3:** Management of patients who had emergency general surgical conditions (*N* = 356)

	*n*	Percent
Non-surgical management^a^		
Antibiotics and other medications	332	93.3
Wound care	177	49.7
Urethral catheterization	36	10.1
Nasogastric insertion	34	9.6
Blood transfusion	7	2.0
Other medical therapy	4	1.1
Overall treatment at district hospital		
Surgery and non-surgical management	185	52.0
Surgery alone	3	0.8
Non-surgical management	143	43.5
No indication of surgery or non-surgical management	13	3.7
For individuals who had surgery at the district hospital (n = 188)		
Hospital of surgery		
Butaro	81	43.1
Rwinkwavu	58	30.8
Kirehe	49	26.1
Type anesthesia (*n* = 110)		
Spinal/Regional	39	35.5
General	33	30.0
Sedation	32	29.1
Local	6	5.4
Operator (*n* = 122)		
General practitioner	74	60.7
General surgeon	48	39.3
Type of intervention (*n* = 186)		
Incision and drainage	114	61.3
Laparotomy	20	10.8
Debridement	17	9.1
Hernia repair	10	5.4
Amputation/disarticulation	9	5.3
Suprapubic catheterization	5	2.7
Skin graft	2	1.1
Biopsy	1	0.5
Others	7	3.8

^a^Possible for patients to have more than one non-surgical management

Of the 145 patients with information on in-hospital post-operative complications, 33.8% (*n* = 49) had at least one complication (Table 4). The most common complication was surgical site infection (13.8%, n = 20) followed by unplanned reoperation (4.8%, *n* = 7) and wound dehiscence (2.8%, n = 4). For 188 patients who received surgery, 85.1% (*n* = 160) were discharged uneventfully, 4.8% (n = 9) were recommended for transfer and 3.2% (n = 6) died. The outcome was unknown for 6.9% (*n* = 13) of these patients. For patients who were discharged (with or without transfer recommendation), the median length of stay was 12 days (IQR = 6, 20). For the 168 patients who did not receive surgery, 39.3% (*n* = 66) were discharged, 35.7% (*n* = 60) were recommended for transfer, 7.1% (*n* = 12) diedand17.9%(*n* = 30) had an unknown outcome. The median length of stay for patients who were discharged without surgery (with or without transfer recommendation) was 8 days (IQR = 5, 14).

**Table 4:** Outcomes for emergency general surgical patients

	*n*	Percent
For patients who received surgery (N = 188)		
In-hospital postoperative complications (*N* = 145)	49	33.8
Surgical site infection	20	13.8
Unplanned reoperation	7	4.8
Wound dehiscence	4	2.8
Cardiac arrest	3	2.1
Unplanned intubation	2	1.4
Urinary tract infection	1	0.7
Deep venous thrombosis	1	0.7
Postoperative complications (others)	11	7.6
Outcomes		
Discharged	160	85.1
Recommended for transfer	9	4.8
Died	6	3.2
Unknown outcome	13	6.9
Length of hospital stay, days (median, IQR) (*N* = 154)^a^	12	(6, 20)
For patients who did not receive surgery (N = 168)		
Outcomes		
Discharged	66	39.3
Recommended for transfer	60	35.7
Died	12	7.1
Unknown outcome	30	17.9
Length of hospital stay, days (median, IQR)(*N* = 65)^a^	8	(5, 14)

^a^Length of stay restricted to individuals who were discharged with or without recommendations for transfer. Patients who died or had an unknown outcome are excluded

In the bivariate analysis, there was a difference in the emergency general surgical conditions (*p* < 0.001) and the type of operative treatment (*p* < 0.001) provided by the general surgeon compared to general practitioners (Table 5). Of the surgeries performed, the general surgeon was more likely to treat acute abdominal conditions, urological emergencies and complicated hernias. In comparison, general practitioners treated more soft tissue infections. Correspondingly, the general surgeon’s surgical load was much higher for laparotomy, hernia repair, and amputation when compared to general practitioner (*p* < 0.001).However, there was no difference in presence of post-operative complication (*p* = 0.332), type of post-operative complication (*p* = 0.222), clinical outcomes (*p* = 0.062) and length of hospital stay (*p* = 0.342) for patients treated by general surgeon or general practitioners.

**Table 5:** Bivariate analysis of patient morbidity, treatment and outcomes if operated on by general surgeon versus general practitioners (*N* = 122)

	General surgeon		General practitioner		
	*n*	Percent	*n*	Percent	*p*-value
Overall diagnosis					
Soft tissue infections	22	45.8	65	87.8	<0.001
Acute abdominal conditions	16	33.3	3	4.1	
Complicated hernia	7	14.6	5	6.8	
Urological emergencies	3	6.3	1	1.3	
Type of intervention					
Incision and drainage	14	29.2	54	73	<0.001
Laparotomy	17	35.4	3	4.1	
Debridement	0	0.0	8	10.8	
Hernia repair	7	14.6	2	2.7	
Amputation/disarticulation	5	10.4	3	4.0	
Suprapubic catheterization	0	0.0	1	1.4	
Skin graft	2	4.2	0	0.0	
Others	3	6.2	3	4.0	
In-hospital postoperative complications (*n* = 110)					
Yes	13	27.7	14	22.2	0.332
No	34	72.3	49	77.8	
Type of in-hospital postoperative complications (*n* = 21)					
Surgical site infection	4	44.5	9	75	0.222
Unplanned reoperation	3	33.3	2	16.7	
Wound dehiscence	0	0.0	1	8.3	
Cardiac arrest	2	22.2	0	0.0	
Outcome					
Discharged	43	89.6	64	86.5	0.062
Recommended for transfer	1	2.1	5	6.8	
Died	4	8.3	1	1.3	
Unknown outcome	0	0.0	4	5.4	
Length of hospital stay, days (median, IQR)(*n* = 103)^a^	42	10.5(8,17)	61	9(5,19)	0.342

^a^Length of stay restricted to individuals who were discharged with or without recommendations for transfer. Patients who died or had an unknown outcome are excluded

## Discussion

In our study, we identified 356 patients with emergency general surgical conditions, with the most common diagnosis being soft tissue infections followed by acute abdominal conditions. This differs from findings reported in other low- and middle-income countries, where acute abdominal conditions are the majority of presenting diagnoses [8, 9]. There are several possible reasons for this difference. First, the results from our study could be a representation of the true burden of disease at rural facilities in Rwanda. Soft tissue infections are generally not referred to tertiary facilities, where many of the previous epidemiological studies have been conducted. As such, soft tissue infections may be underrepresented in these studies’ estimates of the burden of emergency general surgical conditions, which is suggested by a recent study at a Rwandan tertiary hospital that stratified by local and referred patients [10]. Another possible reason is that, given the limited capacity to treat acute abdominal conditions at the district hospital, perhaps these cases are immediately referred from the district hospital to a tertiary facility without proper documentation at the district hospital emergency or surgical units and are thus under represented here.

A second important finding from our study was that patients had symptoms for a long time prior to presentation, especially for soft tissue infections and complicated hernias. Similar delays have been noted for trauma patients in this setting [11]and this is a critical concern as delays in seeking care are linked to poor acute surgical patient outcomes [11, 12]. Studies on health seeking behavior in sub-Saharan Africa have noted that delays to reaching definitive care can stem both from where and when patients seek care and challenges in systems of care [13].These initial delays when combined with referral delays in reaching tertiary hospitals [14] can worsen patient outcomes. Because the majority of patients in our study had health insurance (95% of those with insurance information), which should facilitate access to care, sensitizing communities on the importance of early care seeking behavior, as well as implementing pro-active policies on health system strengthening that support travel to health facilities can minimize these delays [15]. Barriers and facilitators for seeking and reaching care specific to emergency general surgical conditions in rural Africa should be studied further.

Of patients presenting with emergency general surgical diagnoses, approximately half received an operation at the district hospital with operations primarily performed by general practitioners. Due to the shortage of trained surgeons in sub-Saharan Africa, especially in rural areas, surgical procedures are often performed by general practitioners or other trained mid-level cadres [6, 16, 17].However, patients at Butaro District Hospital, the only hospital with a general surgeon in our study, were significantly more likely to receive an operation compared to patients presenting to Kirehe and Rwinkwavu District Hospitals. This is consistent with other studies on surgical delivery in Rwanda [7], and can be attributed to either operations being conducted directly by the general surgeon or by general practitioners supervised by the general surgeon during surgical task sharing [2, 7, 17, 18].In addition, the general surgeon treated the majority of the risky diagnoses (acute abdominal conditions and urological emergencies) and performed complex operations such as laparotomy reflecting improved access to major surgical treatment at a district hospital with skilled surgical provider. Finally, a sizable proportion of patients (45.9%, *n* = 168) did not receive surgery at the district hospital, of whom 57.1% (*n* = 96) were discharged home or their outcome was not reported, revealing potential surgical treatment gaps that largely reflect limited surgical capacity. These findings suggest that it is imperative to recruit and retain surgeons to rural district hospitals, as well as encourage task sharing for non-surgeon providers. Improving access to surgical care at district hospitals by increasing the number of surgical providers, anesthesia providers, and equipped functional theaters will help in advancement of universal health coverage [1, 19].

For patients who did receive surgery at the district hospital, our results also demonstrate that most were discharged without postoperative complications. Secondly, while a substantial portion of our patients did not receive surgery, we were not able to follow-up on their definitive outcome, particularly for those not recommended for transfer. Future studies should prospectively follow such patients to shed light on reasons for their lack of surgical treatment and outcome post discharge. The mortality rate (3.2%) and postoperative complications rate (33.8%) are comparable to studies done in other low- and middle-income settings [4, 8].While there was no difference in post-operative complications, clinical outcomes, and length of hospital stay for patients treated by a general surgeon compared to general practitioners, these outcomes are difficult to compare given the difference in the types of conditions treated. However, this mortality and morbidity can be improved and more studies should be undertaken to understand factors related to poor surgical outcomes to guide efforts to improve the quality of care provided for patients in these settings.

There are limitations to our study that should be considered when interpreting the results. As our study used retrospective chart review, data was missing for some variables due to incomplete documentation in patient charts. A routine audit of patient charts is recommended to encourage complete documentation. Secondly, while a substantial portion of our patients did not receive surgery, we were not able to follow-up on their definitive outcome, particularly for those not recommended for transfer. Future studies should prospectively follow such patients to shed light on reasons for their lack of surgical treatment and their outcomes post discharge. Another possible limitation is the generalizability to the rest of Rwanda or the region, as the hospitals studied are supported by Partners In Health/Inshuti Mu Buzima. PIH/IMB’s support might affect patients’ decision to seek care, as well as patient management in the hospitals. However, these hospitals are still managed by the Ministry of Health, which mandates the same surgical package of care across all district hospitals in Rwanda. Additionally, we do not believe that patient characteristics and disease burden across the rural districts of Rwanda would vary much from what we observed here. Therefore, these results are still relevant to the rest of Rwanda and other settings in rural sub-Saharan Africa.

## Conclusions

Management of emergency general surgery conditions in district hospitals remains a challenge. Our study found that only half of these patients receive surgery. Patients who do receive emergency general surgery at these three district hospitals have low mortality and limited post-operative complications. However, similar to other low- and middle-income countries, there is insufficient trained personnel and infrastructure to address all of the need. Improving surgical capacity at the district hospital and community education for early presentation would likely result in timely, safe and affordable emergency general surgical care at the district hospital and improved patient outcomes.

## Abbreviations

AIDS: Acquired immune deficiency syndrome
HIV: Human immunodeficiency virus
ID: Identification
IQR: Inter quartile range
PIH/IMB: Partners in health/ inshuti mu buzima
US: United States
USA: United States of America
